# “To Suffer in Paradise”: Feelings Mothers Share on Portuguese Facebook Sites

**DOI:** 10.3389/fpsyg.2018.01797

**Published:** 2018-10-01

**Authors:** Filipa César, Patrício Costa, Alexandra Oliveira, Anne Marie Fontaine

**Affiliations:** ^1^Faculty of Psychology and Education Sciences, University of Porto, Porto, Portugal; ^2^Culture, Normativity and Diversity Research Group, Center for Psychology, University of Porto, Porto, Portugal; ^3^Life and Health Sciences Research Institute (ICVS), School of Medicine, University of Minho, Braga, Portugal; ^4^ICVS/3B’s – PT Government Associate Laboratory, Braga/Guimarães, Portugal

**Keywords:** motherhood, feelings, Facebook, cultural model, Portugal

## Abstract

**Background:** Motherhood is an emotional rollercoaster. This is overlooked by most literature, which tends to refer mothers’ pathological states of postpartum depression and anxiety, mainly seeking to understand their causes or predicting factors, and consequences on children’s development.

**Objective:** In this study, we aim to observe the diversity of mothers’ emotional states, and to analyze both positive and negative feelings they disclose on specific public and closed motherhood sites on Facebook. We hypothesize that the intensive motherhood model is prevalent in Portuguese society, thus influencing the type of feelings and circumstances in which mothers disclose them.

**Methods:** We collected posts and comments from the four most popular Portuguese Facebook motherhood sites during 2015 and, then, conducted a quantitative and content analysis to identify the expressed range of feelings concerning motherhood.

**Results:** Mothers preferably share their positive feelings on public pages, whereas negative feelings are shared more in closed groups (CGs). Expressed positive and negative feelings were significantly different whether we look at normative or non-normative, public or closed sites.

**Discussion:** We assume that motherhood sites on Portuguese Facebook reflect an intensive motherhood model that is normative in Portuguese society. Positive feelings toward children are promoted and openly shared in public normative sites, while negative feelings concerning motherhood are dealt with in the privacy of CGs. We propose an extensive motherhood model to overcome this duality and to allow women to pursue several different social roles simultaneously in an equally rewarding way.

## Introduction

Childrearing can be the most gratifying, yet the most demanding, experience of a woman’s lifespan. Culturally, pleasant feelings and fulfillment are attributed to motherhood, which is considered one of the main purposes of every woman’s life. Motherhood is not supposed to be questioned or substituted by any other type of life goals, such as professional achievement, for example. In western countries, namely, in Portugal, even for women who have professional careers, being a mother is still seen as an unavoidable goal that reinforces women’s identities and is associated with positive feelings.

The importance of this primary role over others is socially assumed. An intensive motherhood model is predominant in western societies, a model that assumes the centrality of the child and the prevalence of his/her interests over those of the mother (Elliott et al., 2015; César et al., 2018), who must be completely involved in this role. The mother is expected to nourish strong positive feelings for her child, condensed into the commonly termed “maternal love” (Badinter, 1986). Love and maternal loving dedication are considered not only natural in women, but also essential to appropriate childrearing (Badinter, 1986).

Although motherhood is characterized as an emotional rollercoaster, the absence of positive feelings or the presence of different feelings, such as negative ones, toward motherhood or the child itself are considered unnatural and even pathological. Mothers’ negative feelings and emotional disturbance have been documented from this perspective. Many of these scientific studies about mothers tend to focus on their negative psychological states and/or poor role performance, the respective causes, and predicting factors (Skipstein et al., 2012; O’Hara and McCabe, 2013; Taylor and Johnson, 2013; Tyrlik et al., 2013; Highet et al., 2014; Jover et al., 2014; Agrati et al., 2015; Razurel and Kaiser, 2015; Meier et al., 2016; Tikotzky, 2016; Kim et al., 2017), as well as the consequences it entails for the child’s behavior, development, and well-being (Herba et al., 2013; O’Hara and McCabe, 2013; Jover et al., 2014; Spijkers et al., 2014; Yürümez et al., 2014; Betts et al., 2015; Fairbrother et al., 2015; Junttila et al., 2015; Conners-Burrow et al., 2016; Riva Crugnola et al., 2016; Woolhouse et al., 2016; Granat et al., 2017; Moed et al., 2017). Specific groups, such as teenage mothers (Smith et al., 2017), mothers who experienced preterm labor (Karabekiroglu et al., 2015), or mothers of children who have been clinically diagnosed as disabled (Loukisas and Papoudi, 2016), also arouse scientific curiosity, and are viewed as an abnormal phenomenon, in comparison to normative mothers, assumed as control groups.

Mothers’ positive feelings are seen as necessary and essential for children’s present and future well-being, good development, and behavior, and are only studied in opposition to negative feelings, which cause harm to those same dimensions. We observe that this perspective promotes the prevalence of the child, its needs, and interests, entailing an intensive motherhood model that requires great investment and dedication from mothers, both physical, emotional, financial, and regarding their time (Elliott et al., 2015; César et al., 2018). Spence (2013) refers to a “bonanza built on fear” (p.1), which feeds social media with pregnancy advice, conditions of delivery procedures, compels breastfeeding and induces parents to obsessively control and protect their child, even when real risks are unknown. Summing up, the prescribed mother is happy, emotionally adequate, follows experts’ advice, bonds well with children, and is self-confident. Deviance amplifies self-doubts, and feelings of guilt and shame are induced whenever women, in some way, “fail to live up to ideals of womanhood and motherhood, and (…) transgress cultural expectations regarding feminine modesty” (Taylor and Wallace, 2012, p.76).

When referring to mothers’ feelings, we must specify the meaning. Common sense tends to use the words “feeling” and “emotion” indistinctly, although each word represents a different scientific phenomenon for social and behavioral sciences (Scherer, 2005). To pursue our research, we assume that “emotions are feelings when conscious, and they are not feelings when unconscious” (Prinz, 2005, p.9). Language may help individuals acknowledge emotions, represent it, and more accurately shape it “by contributing to the ability to make situated conceptualizations of emotion in the moment” (Lindquist et al., 2015, p.11). Thus, only emotions that are reflected upon and consciously objectified through thoughts and words are considered “feelings” in our analysis. Since this work is about written language, we considered all words and expressions of overall body sensations, moods, emotions, and/or feelings indistinctively as “feelings,” in the sense that subjects use this form of communication to exteriorize psychological states, even if their exact category within the concept is not determined.

In addition to individual aspects, we acknowledge emotions and feelings as eminently cultural. They are not positive or negative in themselves (Sylwester, 2000), instead they are attributed a positive or negative value by groups and societies according to each values system. Furthermore, “emotions are intrinsically social in that they are typically elicited, expressed, regulated, perceived, interpreted, and responded to in social settings” (van Kleef et al., 2016, p.4). This leads us to agree that a socially considered positive emotional response, feeling, or mood presented by an individual tends to provide social acceptance and psychological well-being (van Kleef et al., 2016), while negative ones elicit worry and even social rejection. By realizing which feelings are considered normative in one context, we also acknowledge which feelings are considered deviant in that same context, since “cultural models involve beliefs as well as social practices that underwrite and sustain what is moral, imperative, and desirable” (Mesquita and Walker, 2003, p.779). Therefore, some feelings and emotional practices are to be promoted or inhibited according to what is culturally valued and accepted. This kind of pressure is created not only by direct social demands, but also by individuals themselves, who internalize social expectations.

In Western societies, the pursuit of personal happiness is considered a positive life goal, and therefore, individuals can comfortably express this feeling. On the other hand, sadness is considered a negative feeling, sometimes even a pathological one. These feelings could also be the result of perceived social acceptance when individuals enact prescribed social roles, such as motherhood. When these roles are achieved in a socially expected way, individuals feel accepted and reinforced, which is associated with positive feelings. However, when this is not the case, negative feelings are elicited. Nevertheless, in a multiple study project, Bastian et al. (2012) found that avoiding the expression of negative feelings did not suppress them: “perceptions of how others evaluate and find acceptable the emotions we experience appear to be an important ingredient in producing downstream emotional responses, and ironically in aggravating those same emotions that are deemed to be socially undesirable or unacceptable” (Bastian et al., 2012, p.78). It is possible to conclude that the non-acceptance of a feeling induces its own reinforcement. Moreover, when the individual tries to inhibit negative emotions such as sadness or is forced to do so, this has potential negative consequences for their mental health (Gross and Levenson, 1997). The stronger the belief about the inappropriateness of a feeling, the “more emotional avoidance and less self-compassion and support-seeking” can be observed in the individual (Sydenham et al., 2017, p.76).

Literature reveals very few qualitative studies on mothers’ feelings through the analysis of their spontaneous, unformatted discourses from a non-pathological and non-normative perspective. Despite the role of new technologies as a powerful means of communication, few studies have focused on their spontaneous use by women, when they face the transition into motherhood. These studies have shown that its use empowers women in their new role, through the sharing of experiences, advice, emotional support, and interaction with other mothers (Drentea and Moren-Cross, 2005; Kaufmann and Buckner, 2014; Neubaum and Kraemer, 2015).

The purpose of this study is to observe the diversity of mothers’ emotional states and feelings about motherhood in everyday life in order to understand how Portuguese mothers are aligned with the intensive motherhood model in their daily life. We also seek to comprehend how Portuguese mothers deal with the emotional demands of the intensive motherhood model, on the one hand, and with denial and pathologizing of mothers’ negative feelings, on the other. We take Facebook motherhood sites as our field of analysis to acknowledge mothers’ main feelings according to their childrearing experience because Facebook is a social network where mothers spontaneously interact and share experiences. We believe that the Internet, particularly social networks, provide enough anonymity for mothers to share and unburden their feelings with a potentially understanding audience, among other functions these sites provide. However, emotional disclosure is also sensitive to the hidden social control and women will only express their feelings if they foresee that they will be understood and accepted. Thus, some differences will be observed among Facebook sites, according to their level of “normativity,” and it is also relevant to analyze which sites encourage or inhibit mothers’ free emotional expression. We aim to analyze (i) which feelings mothers refer to regarding being a mother; (ii) in which situations those feelings arise; (iii) where those feelings are being shared; and (iv) which judgments are made concerning those feelings. We assume these discourses reflect an intensive motherhood model, which is normative in Portuguese society.

We hypothesize that both positive and negative feelings arise in mothers’ spontaneous speeches (*Hypothesis* 1), as motherhood is an emotional rollercoaster; that mothers’ positive feelings are more widely expressed and encouraged on public pages (PPs) than negative feelings (*Hypothesis* 2); in its turn, negative feelings are shared preferably in closed groups (CGs), away from public scrutiny (*Hypothesis* 3a); since these negative feelings are considered deviant in relation to the intensive motherhood model, they are more shared than positive ones in CGs (*Hypothesis* 3b). Finally, feelings, either positive or negative, are qualitatively different in normative and alternative sites: we hypothesize that on the latter mothers share more feelings concerning their own needs and well-being, instead of those of their offspring, which are more common in the normative sites (*Hypothesis* 4).

Facebook motherhood sites were chosen, because it is the most relevant online social network in Portugal, with 98% of Portuguese internet users having a profile on the platform (OBERCOM, 2014). Moreover, studies show that sites on Facebook dedicated to motherhood are places where mothers search for support, advice, and share their experiences and doubts (Kaufmann and Buckner, 2014; Neubaum and Kraemer, 2015), reflecting their values, models, and beliefs regarding motherhood (Madge and O’Connor, 2006).

We believe this is an original and relevant approach, since the literature reveals very few studies regarding mothers’ feelings, and the analysis of mothers’ spontaneous, unformatted discourses seems to be very rare in scientific research. In addition, it is also relevant to analyze the type of feelings institutional sites appear to encourage and/or criticize while addressing posts to follower/member mothers, as they reflect an institutional context that promotes social control.

## Materials and Methods

In a previous study (César et al., 2018), we collected all the Portuguese Facebook sites that were active in 2015, created by or dedicated to mothers, except those focused on selling, advertising, or directed toward overly specific populations. Included were 198 sites, both PPs and CGs, whose descriptions underwent content analysis. We concluded that the majority of Portuguese Facebook motherhood sites promoted the intensive motherhood model, and only on a minority did we find *extensive* (Christopher, 2012) or *negotiated* (Badinter, 2010) motherhood models’ indicators. These latter models advocate that mother and child share protagonism regarding well-being promotion, that the mother reconciles motherhood with other social roles, namely, the professional one, and that other adults may also be considered main caregivers besides the mother, with whom she shares childrearing. For the current study, and given the large amount of information available, we chose a sample of four sites (two PPs and two CGs) with the most followers each to conduct the content analysis. Also, within those four sites we chose representatives of the intensive motherhood model and called them *normative* (PP-normative and CG-normative), and of the extensive motherhood model, which we called *alternative* (PP-alternative and CG-alternative), according to the results of our previous study. In both CG, researchers requested permission to use the information, which was granted as long as the anonymity of the groups and members was preserved (**Table 1**).

**Table 1:** Facebook sample data.

Sites	Months	Followers	Posts	Comments	Total text units	Feelings
PP-normative	12	Over 78,000	594	1931	2525	291
CG-normative	4	Over 1300	819	3818	4637	246
PP-alternative	12	Over 30	42	12	54	9
CG-alternative	8	Over 90	38	97	135	123
Total			1493	5858	7351	669


During 2015, all posts and respective comments from this sample were collected (PP-normative *n* = 2525; PP-alternative *n* = 54). As predicted, normative sites had many more posts and comments than alternative ones, in absolute terms. Since we observed that the information from CG-normative became redundant after merely 4 months, from September to December 2015 (*n* = 4637), we decided to interrupt data collection from this site. As for CG-alternative, the data collection ended after 8 months because the group, created in April, was abandoned in November, after 135 posts.

Public page-normative had more than 78,000 followers in 2015 and it was a blog-style Facebook page that intended to disseminate useful and up-to-date information to parents and future parents on subjects related to pregnancy, newborns, babies, and child development. It had more than 500 links to articles from blogs, in that year, but only text posts were analyzed, as well as readers’ reactions conveyed in almost 2000 comments.

Closed group-normative was a group with more than 1300 followers in 2015, with 819 posts, and its members made almost 4000 comments. Its title suggests it is aimed at parents, though during that year, fathers only made a few comments. The purpose of this CG-normative was to allow its members to discuss and help each other with a range of questions concerning pregnancy, breastfeeding, toys, baby items, child diseases, and post-partum depression.

In the PP-alternative case, in 2015, it had 31 followers, who made 12 comments on its 42 posts. The author considers herself an “unaware” mother who shares her own beliefs and experiences about raising children in a personal and supposedly controversial PP. These ideas are claimed to be based on her motherly instinct and to go against the normativity of childrearing manuals and formal school principles.

The creator of the CG-alternative intended for it to be a non-judgmental place where mothers could share the difficulties of the post-partum period, which are entitled “secrets.” This group focused on the experiences and feelings of recent mothers, rather than on babies or children. Posts lasted only 8 months during 2015, possibly because mothers had passed the puerperium phase and there had been no renewal in its 97 members.

All 7351 posts and comments were uploaded to an NVivo file and a content analysis was conducted to identify feelings mentioned by or about mothers regarding their experience of motherhood. Two first major categories were established: positive and negative feelings, due to positive and negative experiences mothers tend to have (DiPietro et al., 2015). Content analysis was based on the context of full phrases as unit of analysis, through which it was possible to identify the *positive* or *negative* valuation attributed to words (e.g., “surprise” shows up in both valuations) and sentences (e.g., use of sarcasm or irony). Within positive and negative categories, feelings were listed and summed up whenever they occurred. Two other independent researchers reviewed the final lists, with almost total agreement. Throughout the analysis, emergent categories were created. Feelings were considered when expressed either by single words (e.g., “love,” “joy,” “fear,” “sadness”), or by expressions (e.g., “we learn from each other,” interpreted as “solidarity,” “my heart sinks [after scolding her son],” interpreted as “guilt”). Some sets of words with similar meaning (e.g., “tranquility,” “relaxation,” “serenity,” “calmness”) were considered synonyms and gathered in the same category.

In order to test if there were frequency differences of expressed feelings among and within Facebook sites, several chi-square tests were used. We also performed a *post hoc* test using the adjusted standardized residual (AdjSR) to find statistical significant differences between observed and expected counts within cells from the contingency table. Absolute AdjSR values ≥ 1.96 (*p* < 0.05) were considered significant.

## Results

Results show that the four sites under analysis have different functionalities. PP-normative appears to have a pedagogical and regulatory function, as it publishes specialized information concerning motherhood, babies, and childrearing, often written by physical and mental health professionals, who teach mothers how to act and what to feel regarding several matters. CG-normative works as a mutual help group where mothers share their experiences and advise each other based on that same experience. Mothers going through financial difficulties, or those whose children have health, sleep or feeding problems, find other mothers available on this site to provide support and orientation. The small online community of CG-alternative also functions as a mutual help group, although in this case women are able to vent their “secrets,” as well as the unexpected feelings that arise from the transition into motherhood: body transformation, health issues, physical pain, loss of freedom (Highet et al., 2014), marital problems (Highet et al., 2014; Junttila et al., 2015), and difficulties in taking care of a new-born. In turn, PP-alternative presents a very personal perspective on motherhood which shares with CG-alternative the fact that it is not centered on offspring, but rather, it seems to function as a personal diary where this mother publicly shares her ideas and experiences. We did not find any Portuguese sites in 2015 exclusively created by/or addressed to fathers, and we observed an almost total absence of fathers in these sites, even on CG-normative, which is aimed at both mothers and fathers.

We found 699 feelings concerning motherhood in the analysis corpus, 60.7% of which were considered negative ones. This confirms our *Hypothesis* 1 (h1), according to which mothers spontaneously share both positive and negative feelings on Facebook.

Results also show interdependence between types of Facebook pages and expression of feelings [χ^2^(2, *N* = 7351) = 1200, *p* < 0.001].

**FIGURE 1:**
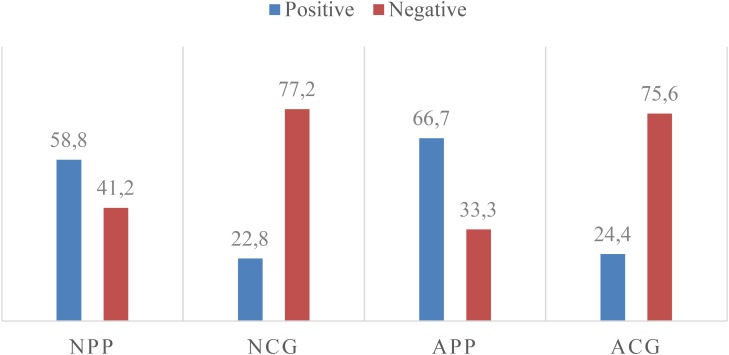
Positive and negative feelings per site (%).

Significant differences between PPs and CGs concerning the occurrence of positive and negative feelings were observed. Positive feelings were more frequent in PPs (67.3% of all positive feelings; AdjSR = -9.4), which confirms *Hypothesis* 2 (h2), while negative feelings were more frequent in CGs (69.7% of all negative feelings; AdjSR = 9.4), confirming *Hypothesis* 3a (h3a) [χ^2^(3, *N* = 699) = 88.4; *p* < 0.001]. Moreover, within CGs, either normative or alternative, expression of negative feelings is more frequently shared than positive ones [χ^2^(1, *N* = 699) = 68.4; *p* < 0.001], thus confirming *Hypothesis* 3b (h3b). In fact, 77.2% of feelings expressed on CG-normative are negative (AdjSR = 6.7), and in CG-alternative, they represent 75.6% (AdjSR = 3.8; **Figure 1**).

Positive feelings’ encouragement appears on both normative sites, instead of exclusively on PPs, as we hypothesized (h2). Either in PP-normative and in CG-normative, positive feelings toward children and motherhood are not only openly shared by mothers [“It is unspeakable to be able to love so much and so strongly two such special beings at the same time!!,” “Normal parents (…) protect, care for and love their children,” CG-normative], but also promoted and encouraged, including by professionals on PP-normative (psychologists, nutritionists, and nurses, among others), which only partially confirms our second *hypothesis* (h2): as state by Developmental Psychologists in PP-normative, “Ignoring the baby’s crying goes against the natural parental instincts of loving, caring and being there for our babies,” “… introduction of solids! Let’s make this first meal enjoyable and relaxing for everyone,” or by mothers in CG-normative “A young child needs a lot of love and a lot of spoiling.”

On the other hand, negative feelings are received with support and encouragement by other mothers only in CGs, as showed by *solidarity*. Differences between the four sites were found [χ^2^(3, *N* = 263) = 68.4; *p* < 0.001] concerning this feeling. In PP-normative, solidarity is completely absent (AdjSR = -5.2), while in CG-alternative, it is what mothers show the most among themselves (36.7% of its positive feelings; AdjSR = 8.1). Here, they share their experiences: “These are real testimonies that actually help,” “I understand it so well...,” “Your words are indeed comforting! Not that I’m happy to know that I’m not the only one to go through this less good phase, but because I can talk to people who understand me.” Solidarity is rarely present in CG-normative (only three references), although its mothers seem to form a real online community: “Sometimes we get desperate because we think that difficult things only happen in our home and it turns out that it is something that happens to everybody! And we learn from the solutions that other mothers suggest.” Significant is the fact that we didn’t find solidarity in either PP. Nevertheless, it cannot be assumed from mothers’ testimonies that their negative feelings go against the intensive motherhood model, but only that they struggle to perform the prescribed role successfully. In fact, some of them refer to that same intensive model, as will be discussed below. Thus, *Hypothesis* 3b (h3b) cannot be fully confirmed.

Indeed, we also expected that feelings expressed by mothers would be qualitatively different in normative and alternative sites (*Hypothesis* 4). In order to test this *hypothesis*, deeper analyses of the meaning of positive and negative feelings were performed.

### Positive Feelings

With regard to positive feelings, the most reported one is *love* toward infants, with 99 references (37.6% of all positive feelings; **Table 2**). It is mentioned in CG-normative (32.1%), as expected by chance, but it is more frequently mentioned in PP-normative (46.2% of its positive feelings; AdjSR = 3.7) and never in CG-alternative [AdjSR = -4.3; χ^2^(3, *N* = 260) = 22.280; *p* < 0.001]. Love is mentioned for various reasons, which allowed some different meanings to emerge from the analysis. Not only is it a very strong feeling, (“It is unspeakable to be able to love so much and so strongly two such special beings at the same time!!,” CG-normative; “The love of a mother can only be compared to the love of God: pure, true and powerful,” PP-normative; “unconditional love,” PP-alternative), but it is also seen as a “natural” one (“Even animals take better care of their baby because they act with instinct, with love,” PP-normative), as well as a mother’s obligation (“Our job is to transmit love and confidence!,” PP-normative; “our role as parents it’s not to teach: it’s to love!,” PP-alternative). Love is also fundamental for good child development (“Lack of attention and love from parents is what causes bad manners,” PP-normative), and it is a strategy to deal with tantrums (“Calm down and [give her] lots of love!,” PP-normative).

**Table 2:** Positive feelings.

	PP-normative		CG-normative		PP-alternative		CG-alternative		Total	
	*n*	%	*n*	%	*n*	%	*n*	%	*n*	%
Adoration	1	0.6	1	1.8	0	0	3	10.0	5	1.9
Affection	28	16.4	3	5.4	0	0	1	3.3	32	12.2
Calmness	10	5.8	3	5.4	0	0	1	3.3	14	5.3
Comfort	1	0.6	0	0	0	0	2	6.7	3	1.1
Comprehension	1	0.6	0	0	0	0	0	0	1	0.4
Dazzle	3	1.8	4	7.1	0	0	0	0	7	2.7
Empathy	1	0.6	2	3.6	0	0	0	0	3	1.1
Expectation	4	2.3	1	1.8	0	0	0	0	5	1.9
Freedom	0	0	0	0	0	0	1	3.3	1	0.4
Fulfillment	2	1.2	1	1.8	0	0	2	6.7	5	1.9
Gratification	3	1.8	2	3.6	0	0	0	0	5	1.9
Happiness	9	5.3	3	5.4	1	16.7	1	3.3	14	5.3
Hope	1	0.6	2	3.6	0	0	1	3.3	4	1.5
Joy	1	0.6	1	1.8	0	0	0	0	2	0.8
Love	79	46.2	18	32.1	2	33.3	0	0	99	37.6
Perseverance	2	1.2	1	1.8	0	0	1	3.3	4	1.5
Pleasure	7	4.1	2	3.6	1	16.7	1	3.3	11	4.2
Pride	5	2.9	3	5.4	2	33.3	1	3.3	11	4.2
Relief	3	1.8	1	1.8	0	0	0	0	4	1.5
Resistance	0	0	0	0	0	0	1	3.3	1	0.4
Self-confidence	7	4.1	3	5.4	0	0	1	3.3	11	4.2
Solidarity	0	0	3	5.4	0	0	11	36.7	14	5.3
Surprise	0	0	1	1.8	0	0	2	6.7	3	1.1
Tenderness	1	0.6	1	1.8	0	0	0	0	2	0.8
Trust	2	1.2	0	0	0	0	0	0	2	0.8
Total	171	100	56	100	6	100	30	100	263	100


Close to love and sometimes in association with it, *affection* is also more referred in PP-normative (AdjSR = 2.8), its second most reported feeling, than in other sites, where this reference is absent or scarce (AdjSR < 1.96), [χ^2^(3, *N* = 263) = 8.3; *p* = 0.04]: “babies only want love and affection”; “The best thing is to give them the Love and Affection of our permanent presence”; “affection is everything…” On the CG-normative site, it mostly relates to the willingness to cuddle, kiss, and hug babies and children: “I really want you to know that my chest will always be yours, my lap will always be yours, that my arms will always be opened and that my love will never fade.” If we join love and affection, as the most usual physical manifestation of love, it is observed that they represent 49.8% of all positive feelings mentioned in the four sites, and to 62.6% in the case of PP-normative.

However, love or affection for children is completely absent in CG-alternative. Instead of this, here we find *adoration* (10.0%) (AdjSR = 3.5) [χ^2^(3, *N* = 263) = 12.3; *p* = 0.007] about being a mother and breastfeeding, referring to mothers’ own pleasure, a feeling that is lower than expected in PP-normative (AdjSR = -2.1). This confirms our *Hypothesis 4* assumption that mothers share more feelings concerning their own needs and well-being in alternative sites. Only PP-alternative does not confirm *Hypothesis* 4 as far as love is concerned (AdjSR = -0.2).

Mothers from the four sites also feel *happiness* (5.3% of all positive feelings) associated to the experience of motherhood. In this particular case, *Hypothesis* 4 is not confirmed as there is no clear difference between normative and alternative sites [χ^2^(3, *N* = 263) = 1.8; *p* = 0.622]. Instead, CGs’ mothers show some similarities in their discourses when they say they feel happy despite the difficulties, as if they were worth it: “I am very happy to be the mother of two wonderful beings”; “A child changes everything! Even the mother! (…) /10-things-that-change-radically-whith-children/ (…) [Comment #1:] Fantastic! Seeing it all written like this, so tidy, and seeing that everything is the deepest truth... and that [it] has brought us the greatest happiness in the world,” PP-normative. Nevertheless, both CG also refer to mothers’ happiness despite difficulties: “But the greatest strength is to wake up every day and look at my little boy and see that all those clichés are really true and there is nothing in the world that makes me happier!,” CG-alternative; “It will work out, you’ll see… it’s hard work, but you get a lot of pride and happiness too”; “It’s essential that people respect the baby and parents, that we stop judging each other, that we accept that each baby is unique, and that each family is unique. I have no doubt that it’s the formula for success for a happy parenting, for happy babies,” CG-normative. In PP-alternative, happiness is also felt both by the mother and children: “that makes us very happy.”

*Calmness* (4.0% of all positive feelings) is referred in almost all sites as the key to enjoy a more gratifying motherhood [χ^2^(3, *N* = 263) = 5.2; *p* = 0.157]. PP-normative psychologists or other specialists even promote it to benefit both mother and children: “One day your child will begin to eat solid foods! Let’s make this first meal enjoyable and relaxing for everyone”; “Advice for the first time with your baby to be as calm as possible”; “[Here is] A practical, uncomplicated book that helps parents / educators find a more serene and happier way to raise their children, without shouting and without spanking!”; “My motto is: Relax and let nature do the rest! Parenting is wonderful, do not ruin it with so much theory.” CG-normative mothers also try to promote their own and their children’s serenity: “It was hard for me when she went to day care, I felt like a bad mother, but then I realized that we are all happier this way: Mom can work and be herself, and she can also be a mother, and we are all calmer and more serene and so we enjoy more of each moment”; “When I let him choose [his food] and when I avoided giving him what he doesn’t like, and I noticed that if the soup had “little balls” he would eat... that was my rest!”; “My princess used [a baby item] so much, and I was relaxed.” We find no meaningful differences in CG-alternative mother stories: “Everything went super well! The mouth, the milk coming in and even the milk drying up now at 9 months! I think the fact that I felt supported gave me a lot of relaxation and it was all natural!.” Thus, calmness also invalidates *Hypothesis* 4.

### Negative Feelings

But most feelings we found are negative (60.7% of total references), and these also appear in more diverse forms (25 types of positive feelings vs 28 types of negative feelings). We will present doubt, effort, and suffering, which are the ones most mentioned (**Table 3**).

**Table 3:** Negative feelings.

	PP-normative		CG-normative		PP-alternative		CG-alternative		Total	
	*n*	%	*n*	%	*n*	%	*n*	%	*n*	%
Anguish	4	3.3	7	3.7	0	0	0	0	11	2.7
Anxiety	2	1.7	1	0.5	0	0	2	2.2	5	1.2
Depression	2	1.7	5	2.6	0	0	5	5.4	12	3.0
Despair	5	4.2	8	4.2	0	0	3	3.2	16	3.9
Disaffection	0	0	0	0	0	0	1	1.1	1	0.2
Discomfort	2	1.7	0	0	0	0	3	3.2	5	1.2
Distrust	0	0	3	1.6	0	0	0	0	3	0.7
Doubt	33	27.5	38	20.0	0	0	2	2.2	73	18.0
Effort	13	10.8	22	11.6	1	33.3	7	7.5	43	10.6
Fear	5	4.2	25	13.2	0	0	1	1.1	31	7.6
Frustration	1	0.8	5	2.6	0	0	3	3.2	9	2.2
Fury	0	0	1	0.5	0	0	1	1.1	2	0.5
Guilt	3	2.5	2	1.1	0	0	1	1.1	6	1.5
Insecurity	2	1.7	3	1.6	0	0	3	3.2	8	2.0
Loneliness	0	0	0	0	0	0	8	8.6	8	2.0
Loss	1	0.8	4	2.1	0	0	8	8.6	13	3.2
Missing	4	3.3	6	3.2	0	0	0	0	10	2.5
Pity	3	2.5	5	2.6	0	0	0	0	8	2.0
Regret	0	0	0	0	0	0	1	1.1	1	0.2
Resentment	0	0	2	1.1	0	0	13	14.0	15	3.7
Sadness	0	0	3	1.6	0	0	3	3.2	6	1.5
Stress	2	1.7	8	4.2	0	0	4	4.3	14	3.4
Suffering	17	14.2	14	7.4	2	66.7	10	10.8	43	10.6
Surprise	0	0	0	0	0	0	3	3.2	3	0.7
Terror	1	0.8	2	1.1	0	0	3	3.2	6	1.5
Tiredness	8	6.7	14	7.4	0	0	5	5.4	27	6.7
Uncontrolled	1	0.8	0	0	0	0	3	3.2	4	1.0
Worry	11	9.2	12	6.3	0	0	0	0	23	5.7
Total	120	100	190	100	3	100	93	100	406	100


Mothers tend to share their *doubts* more in normative sites (PP-normative: 27.5%; CG-normative: 20.0%) [χ^2^(1, *N* = 406) = 21.5; *p* < 0.001] (AdjSR = 4.6) concerning several decisions, lack of knowledge, and insecurities they keep facing while performing their role. These doubts relate to general care, health and diseases, child development, child feeding, sleeping and co-sleeping, going to day-care, and managing different opinions: “I was also advised in the birth preparation sessions not to use alcohol, but the truth is that many of my colleagues, mothers, recommend the use of 70° alcohol at... Contradictory opinions that leave us indecisive”; “We always have some doubts about the development of our children, whether they are within normal development parameters or not”; “My baby is 10 months old and doesn’t drink water... what can I do to get her to start drinking? I give it to her but she pushes it away”; “My baby is 1 month old, and I’m kind of lost with his instability to sleep, and this text made a few things clear!”; “Today was also the first day of my [baby’s name]. She will be 12 months on the 11th. I didn’t want to put her [in day-care] but I have no alternative... she didn’t cry when I left her there but she cried in my absence and I just left her for 1 h… Is it possible to leave them like this? Do you think there’s no problem? Doesn’t it affect her personality? She is such a brave, lively baby, but there she seemed so ‘little,’ so forgotten…”; “My biggest problem is how does she feel? Because she looked abandoned, she was sitting next to the teacher, but she was confused, half lost, as if I was horrible to have left her there... the separation is horrible, but I can deal with that, my problem is her! Is it really the best for her? Is she crying a lot? Does it hurt her? Is she scared?”; “The first [soup] I made with potatoes, pumpkin, onion, and a little of olive oil. My little one loved it. I have a question with regards to greens and garlic. The article says that it should be introduced after 12 months. Meanwhile at the health center they said that I can already give them to my baby. She’s 5 months old... now I’m worried. Should I give them or not?,” PP-normative; “Our mothers were toxic, our mothers-in-law were even worse... and not wanting to be like them, do we do better or worse? Doubt causes great anguish!”; “My question is that the fever never goes down from 37.7° and when it starts to increase it always goes to higher values, is this normal? Is there no infection here? How high is it safe for me to continue with this treatment?”; “I’m going to give diversified food to my baby and I’d like to know how you freeze the soup pots or vegetable purees. Whether you make it hot or cold. And how do you make vacuum, so bacteria don’t get in?”; “Let’s see, we have here two pediatricians with opposing guidelines, I gave [my child] the 1st dose because my pediatrician also recommended it, but this week I saw the news (...) about the deaths related to the vaccine, certainly all drugs have contraindications and side effects, but Kawasak Syndrome is very serious, and I was reading about it and what I saw was that the ratios corresponding to the vaccine and to the syndrome are highly different, i.e., the mortality rate is higher with the Kawasak’s syndrome than with meningitis. Confused confused confused !!!!,” CG-normative.

PP-normative also attempts to enlighten parents on many subjects, divulging testimonies of professionals: “When a baby is born, doubts and more doubts arise in parents, especially in first-time parents. We put 6 together, the most common in the first few weeks of your baby”; “We collected the main doubts we are asked about the little ones’ healthcare and we went looking for answers!”; “Backpacks are part of the daily routine of all school-aged children, and all parents have come across this question: give in and buy what they like, or look for the best in terms of ergonomics? And then comes the question by Physical Therapist: “But how do I know what’s best in terms of ergonomics, knowing so little of the subject?.”

On alternative sites, only CG-alternative mothers express doubts concerning breastfeeding: “Questions began to arise, and no one advised or helped me in the hospital.” But references on alternative sites are scarce and not specifically related to children’s well-being, as they are on normative sites. Therefore, we assume that Doubt is a feeling in which *Hypothesis* 4 is confirmed.

On all sites (PP-normative: 10.8%; CG-normative: 11.6%; PP-alternative: 33.3%; CG-alternative: 7.5%) [χ^2^(3, *N* = 406) = 2.8; *p* = 0.429], mothers claim to put great *effort* into their activity, which they claim is difficult, complicated, or physically and emotionally hard: “My biggest difficulty is the part of her falling asleep alone in her bed. She cries so much that I end up putting her to sleep on my lap and then I lay her down on the bed”; “Actually postpartum is not easy, breastfeeding, episiotomy, bad night’s sleep, it’s survival...”, PP-normative; “I would love to see him eat anything... but until now it has always been very complicated”; “I know it’s not easy but we get strength where we cannot imagine,” CG-normative; “As they grow older, other difficulties arise, but I think we see it differently because we become hardened”; “If I knew it was going to be difficult? No, but I imagined, [did I know] on what scale it would the difficult? I really didn’t know,” CG-alternative; “To be a mother (or father) is to be, necessarily, someone who works behind the scenes. Our names are those that no one reads in the final credits. It wouldn’t be easy to be the sound technician on the BFTA’s night, the Pulitzer editor, or the mother of the Nobel Prize for Chemistry. But as my children say, ‘you’re the one who wanted to be a mother!’,” PP-alternative. All references to effort are balanced across the four sites, which does not sustain *Hypothesis* 4.

*Suffering* is also mentioned on all four sites (PP-normative: 14.2%; CG-normative: 7.4%; PP-alternative: 66.7%; CG-alternative: 10.8%), but more frequently in PP alternative (AdjSR = 3.2) [χ^2^(3, *N* = 406) = 13.7; *p* = 0.003]. It includes words related to the verb “to suffer” and to situations where mothers refer to real pain. On PP-normative, suffering is associated with sleeping problems and leaving babies at day-care: “To be a mother, is to suffer in paradise!”; “It hurts very, very much, they are so small and so helpless, they need their mother so much”; “I have suffered so much for each one differently.” CG-normative mothers also complain about chores and children’s problems related to health, food, and behavior: “There are both sides, the pain of not having done anything and something happened, or the pain of doing something and it goes wrong...”; “I’ve been told to let her decide whether to eat or not, but I can’t! I always insist because if she decides, she can spend days without eating or eating almost nothing, and she is already small. Mothers suffer!!”; “[my son] had a sleep disorder, even took melatonin (12 drops only to fall asleep, it didn’t prevent him from waking up after 2 h).... He suffered... We suffered!!!!”. Also, what makes a PP-alternative mother suffer is her children’s suffering: “nothing hurts me more than having a sick or hurt child.”

In CG-alternative, mothers’ suffering is associated to the post-partum period, breastfeeding, and hearing criticism: “I’ve heard a lot of things during my first months of motherhood, and even now, and sometimes it hurts”; “No course told me that breastfeeding would hurt so much”; “I almost gave up breastfeeding my son, tears rolled down my face and it was a martyrdom!.” Again, although CG-alternative only refers to mothers’ well-being, this concern is also present in normative sites, and the PP-normative mother is mainly concerned with her children. Again, these results do not sustain *Hypothesis* 4.

However, it is noteworthy that some specific negative feelings are exclusively found on some of the sites under analysis. These specificities may be considered at least a partial confirmation of *Hypothesis* 4.

The most referred feeling among CG-alternative’s mothers is *resentment* (14.0% of its negative feelings) (AdjSR = 6.0), comparing with both normative groups (AdjSR = -2.6) [χ^2^(3, *N* = 406) = 36.1; *p* < 0.001] toward everyone who failed to warn them about what was coming with motherhood, and toward excessive advice: “I wish I had been told a lot of things in difficult times... It helps to overcome insecurity, the feeling that we are the worst mothers in the world, that everyone knows except for us”; “the worst are those first months where everything happens at the same time without any preparation!!”; “That when I saw the baby for the first time I wouldn’t fall madly in love”; “That I would cry and feel such a great sadness in the early days. And the weeks to follow. And that this was all normal (damned hormones) and that I was not a bad mother because of that”; “When I needed encouraging words I only got them from my family. I had a cesarean and only managed to breastfeed for 1 month, and that made me the target of the most varied criticism (…) we are such ‘bitches’ to other people. Luckily, I didn’t have any psychological breakdown, as in ‘baby blues,’ but only imagine the wickedness that can come from a simple comment”; “I think there are ‘others’ as guilty or worse than hormones and everything we read... The advice we are told!!! Everybody knows better than us why our baby cries, why he doesn’t gain [weight], why he regurgitates, why he doesn’t sleep... It’s terrible!!!!.” This feeling can also be found in CG-normative, albeit in a lesser proportion: “They say it’s all a bed of roses... it was nothing like that and I just felt like I wasn’t ready to be a mother...”

Regarding CG-normative, most mothers refer to a constant *fear* (13.2% of its negative feelings) (AdjSR = 3.9) contrary to what happens in CG-alternative (AdjSR = -2.7) [χ^2^(3, *N* = 406) = 16.2; *p* = 0.001]. CG-normative mothers are afraid that something bad could happen or go wrong with their children, such as accidents, sickness, or abductions: “I’m afraid, and I prefer him to sleep in the crib, but I also take care of him in the crib so that there’s nothing within his reach that can do any damage”; “She slept with me about three times and then I started to get scared that she would get hurt and started to lay her on her bed”; “I’m afraid that at night the fever rises more than 39.9 as it was a while ago...”; “I’m a ‘panicker’ and I go straight to the hospital! Or I annoy the pediatrician!”; “I’m scared you know?!?! Falls and small accidents are part of life for me, and when they have to happen, that’s the learning process, it’s part of life. Now, to lose them or if someone takes them... panic!”; “I am very fearful and I am always very, very afraid of what might happen to them... at their age I already used to go out to buy bread, and mine [only went twice] (…)... I see the grocery store from home, but so what? If someone passes by and takes them, I can only watch it, I’m not a flash to go down four floors in seconds, so... they leave [home] with me or don’t leave at all.”

Finally, only on normative sites can we find *Worry* in mothers’ speeches (PP-normative: 9.2%; CG-normative: 6.3% of their negative feelings) [χ^2^(1, *N* = 406) = 7.6; *p* = 0.006] (AdjSR = 2.7), concerning their children’s health, sleep, global development, feeding, and safety. This feeling is absent in alternative sites. PP-normative institutional posts attempt to address parents’ worries, providing information that could enlighten and eventually reassure them: “Child obesity, bullying, drugs and Internet safety head the list of top 10 concerns of American parents. What is your biggest concern about your child’s growth?^1^”

Two other feelings have more occurrences in CG-alternative than in other sites: *loneliness* and *loss* (8.6% of its negative feelings, each) (AdjSR = 6.3) [χ^2^(3, *N* = 588) = 40.7; *p* < 0.001]. They are less referred than expected both in CG-normative (1.6%; AdjSR = -2.2) and PP-normative (0.5%; AdjSR = -3.0). CG-alternative is the only site where mothers confess feeling lonely despite spending time with their children. It comes from the absence of a social life or the separation from friends who are not parents: “Sometimes I miss my social side. I don’t have many friends with children and at this moment of my life I feel that I only really know how to be a mother and all the conversations revolve around this, I’m probably boring... Okay, I just wanted to get this off my chest”; “I found the first few months very lonely indeed, and especially the nights... my husband was snoring like a pig and I had to put up with the endless feeding every 2 h...” Loss is related to social life, mother’s freedom, and previous body: “We all feel this with our first child because it’s the loss of freedom, and a great change in life”; “Cellulite, stretch marks and extra pounds, hurting breasts that cannot fit in any bra were not enough, now I also had to go bald?!!!”; “From one day to the next, we stop sleeping, we cannot go to the bathroom, we have absolutely disproportionate breasts (…), stitches whether it was a normal birth or a cesarean... The sofa is over, meals at decent hours are over, if there are meals at all.”

## Discussion

Facebook and other online platforms may be considered an important way to seek social support for mothers, who are able to find numerous sites that meet their different needs and experiences. Analyses of mothers’ feelings on Facebook sites performed in this study allow us to conclude that motherhood brings a range of positive and negative feelings, which are differently shared, welcomed and valued on different Facebook sites. This confirmation of our *Hypothesis* 1 (h1) sustains the perspective of motherhood as an emotional rollercoaster.

Since motherhood is socially believed to provide mothers with personal fulfillment and pleasant feelings, and experiencing such positive feelings is considered publicly disclosable, as sustained by our *Hypothesis* 2 (h2), results confirm that more positive feelings are shared in PPs. But when perception of inadequacy to meet the intensive motherhood model is experienced, arousing negative feelings, it appears that women seek support in CGs, which seem to function as mutual aid groups (h3a and h3b). This enhances the importance of social support during different stages of motherhood (Skipstein et al., 2012; O’Hara and McCabe, 2013; Jover et al., 2014; Razurel and Kaiser, 2015). Another possible interpretation is that the privacy of CGs protects mothers from public exhibition and consequent scrutiny in case their feelings are less socially accepted.

Interaction between mothers is lower in PP-normative, particularly in the proportion of publications and comments by number of followers or members, possibly because this page has a more informative or “educational” function, with mothers assuming a more receiving role. Nevertheless, PP-normative is a much more frequently consulted site than any CG, and it is a place where mothers can confirm if their performance matches to the norm or learn to improve it. In both cases, we observed mothers’ perceived empowerment, also reported by Drentea and Moren-Cross (2005), Kaufmann and Buckner (2014), and Neubaum and Kraemer (2015).

Qualitative differences among feelings were observed on the four Facebook sites according to their more normative representation of motherhood or their openness to an alternative model, sustaining our *Hypothesis* 4 (h4). Indeed, positive feelings on normative sites are almost exclusively focused on the children (love, affection, tenderness, pride), which is also consistent with the intensive motherhood model, requiring maternal love and considering it not only essential, but also natural (Badinter, 1986). Reification of maternal love agrees with literature that considers the mother’s attachment as mandatory (Cooke et al., 2016) as its absence entails risks for the child’s development (Yürümez et al., 2014; Fairbrother et al., 2015; Junttila et al., 2015; Riva Crugnola et al., 2016). The possibility and importance of other attachment figures besides the mother, or childrearing responsibility by the father, other family member, or the community, are never (or rarely) mentioned. Even without testimonies from fathers, we can infer from the mothers’ testimonies that meaningful gender differences in family tasks remain, and that mothers are the main caretakers and assume a set of responsibilities in childrearing, particularly at home (food, hygiene, sleep, and health). Indeed, the literature supports the gender division of family work, both in Portugal and abroad, but some changes are enhanced as fathers start to participate more in childrearing. Fathers do not assume as many tasks and responsibilities for childrearing as the mothers, nor do they feel comfortable sharing their new experiences, at least on Facebook, or both situations.

Negative feelings mentioned on normative sites relate mostly to doubts mothers have concerning childrearing and the effort it requires. Here, mothers disclose great concerns about the best way to act in every circumstance, how to respond to the child’s every need, and how to avoid doing something “wrong” (doubt, suffering, effort, worry, fear). These feelings also stem from the intensive motherhood model (Elliott et al., 2015), which requires total commitment from mothers, exclusively centered on children’s needs and well-being. Embracing such a demanding model contributes to mothers’ insecurity and performance anxiety (Skreden et al., 2012; Taylor and Johnson, 2013; Jover et al., 2014; Offer, 2014), which are expressed in the fear of “doing harm” or “traumatizing” children by introducing a new food too soon, or by leaving them crying at the day care, as we have seen in normative sites. Therefore, intensive motherhood seems to be promoted and reinforced through normative Facebook sites like PP-normative, where mothers’ positive feelings are encouraged and where they can (i) learn how to act “correctly,” (ii) confirm that they are “correctly” performing their tasks and/or that their offspring is “correctly” developing, (iii) or, if not, they know the best way to “correct” themselves, according to that model. Mothers using normative sites express great concerns about being a “good” mother, which is reflected on CG-normative through the fear of drifting away from that ideal. They focus on their children’s well-being, associated with the penalty of constantly feeling worry and lacking confidence (doubt). Self-denial is evident, even if fatigue is recognized. Yet, socially, these feelings are not considered a real problem, as they seem to be inherent to intensive motherhood and this is, thus, the effort every mother needs to make in order to have healthy and happy children.

On the other hand, on CG-alternative children are not the central issue. Here, both positive and negative feelings tend to concern mothers themselves or are directly related to them. These feelings include adoring being a mother or breastfeeding, and perplexity and resentment toward the way motherhood has been socially conveyed, without prior, full information about how hard it would be for them and their bodies. Moreover, here solidarity arises as the most mentioned positive feeling, and derives from finding a place where new mothers can share their negative feelings and health issues without being negatively judged. Solidarity only arises in CGs, and more frequently in the alternative one. In normative sites, mothers and professionals provide advices and solutions so that other mothers get closer to the intensive motherhood model – which does not happen in CG-alternative. Here mothers react to a new reality they were not warned about and confirm that their issues, that include feelings loneliness and loss, are indeed quite common. According to Gross and Levenson (1997), the possibility of expressing these unexpected negative feelings can be considered essential to mothers’ mental health.

However, some feelings were found in both normative and alternative sites, such as happiness, calmness, effort, and, to some extent, suffering.

Public page-alternative site seems to be open to both views of motherhood: being a public site, mother’s positive feelings are closer to the normative pattern (love and pride toward offspring), while her negative feelings follow the alternative model, since she mentions her own effort and suffering. So, PP-alternative can be considered an exception, as it provides a context where a mother can dedicate herself to her children intensively, while openly speaking about the demands of motherhood and the loss of other life dimensions.

As can be seen by the above, considering the Facebook sites under analysis, and taking into account that they represented its most popular normative and non-normative pages and groups in Portugal in 2015, we can conclude that PPs on Facebook mainly disseminate the intensive motherhood model (i) enhancing the centrality of the child and its well-being, on public and normative sites; (ii) by leading the expression of negative feelings it generates to CGs, which emerge from that centrality and from the demands concerning maternal performance; and (iii) by the difficulty in publicly expressing unease with regard to issues about motherhood and women’s identity – which are disclosed hidden from public scrutiny, as if they were deviant themes or expressed deviance to the norm.

Summing up, negative feelings can be divided into *disclosable* and *concealed*. Disclosable negative feelings arise from the demands of the intensive motherhood model (doubt, worry, fear) and can be related to anxiety, performance, effort to become a “good” mother, to not harm the child’s present or future in any way, to properly do what is socially expected. These are mainly expressed in CGs. Concealed negative feelings arise from a set of unexpected changes to women’s bodies and lives, with consequences for their physical and mental well-being, about which people do not openly talk. Resentment toward lack of information demonstrates that women have not been prepared for these types of feelings and situations. On the other hand, keeping them private will contribute to their accentuation and continue this negative cycle, with repercussions on mothers’ well-being (Gross and Levenson, 1997; Sydenham et al., 2017). CG-alternative will play an important role in keeping this situation more visible and acceptable, providing mutual support.

In contrast with the scarce literature on the impact of mothers’ negative feelings on their future quality of life, significant research focuses on the development of children and on the impact of maternal practice on the early years of life and on their future – which contributes to consolidate the intensive motherhood model and increases social and internal pressure on mothers. Nevertheless, there are no studies on the medium and long-term effects of such a pressure on women’s lives and on their future well-being.

We believe that the disclosure of concealed negative feelings would have an important role in changing the way society views parenthood, in enhancing the importance of mothers’ well-being beyond the mother–child relationship, and in considering serious difficulties associated with motherhood. Bringing these two sides of the coin together – gratifying and penalizing – in an open and enlightening way, would allow women to be more aware of the implications of motherhood and, consequently, make more informed choices during their lifespan. Furthermore, the promotion of a less intensive motherhood model – an extensive one (Christopher, 2012; César et al., 2018), less child centered, more dedicated to mothers’ needs and well-being (logistic, physical, emotional...), where childrearing and nurturing are tasks shared with other adults and not only the mainly mother’s responsibility – could transform motherhood into an experience which causes less anxiety and requires less effort. Moreover, a socially approved extensive motherhood model would be more compatible with other domains of women’s lives, such as social and professional domains, thus allowing them to fully experience their different social roles and potentially not feel the need to postpone or even abandon the decision to have children, seen as a project which is too demanding.

The fact that this study only covers Facebook users during a year is a limitation. Yet, the large amount of information available made it possible to analyze only four sites, which could have been chosen using different criteria. Regarding CGs, the sample was chosen from those who authorized the entry of the researcher and, subsequently, those who authorized the anonymous use of data. Also, CG-alternative site members constitute a specific population of new mothers going through a particularly difficult period of motherhood. Nevertheless, we considered it a pertinent strand for our theme, along with other strands that could serve the same purpose. In fact, this is also the case of many followers of the other sites. In addition, the impossibility of distinguishing sociodemographic variables (among others) from followers/members prevented us from taking the results of this research further.

## Ethics Statement

All information used in this study respects the anonymity of all sites’ titles, users and administrators. In the case of the two closed groups, the content was used with their administrators’ informed consent.

## Author Contributions

This research and this article were developed by FC during her Ph.D. studies, both under the supervision and with the contributions of AF and AO. Statistical tests and analysis were performed by PC and AF.

## Conflict of Interest Statement

The authors declare that the research was conducted in the absence of any commercial or financial relationships that could be construed as a potential conflict of interest.

## Footnotes

1 http://www(...)/-the-10-major-parents-concerns
